# Understanding volunteer motivations and concerns in coaching and officiating basketball: implications for sport policy

**DOI:** 10.1186/s12889-023-15949-5

**Published:** 2023-05-25

**Authors:** Meghan Casey, Jack Harvey, Melanie Charity, Scott Talpey, Lindsey Reece, Rochelle Eime

**Affiliations:** 1 Institute of Health and Wellbeing, Federation University Australia, Ballarat, Australia; 2 SPRINTER Research Group, Prevention Research Collaboration, Charles Perkins Centre, University of Sydney, Sydney, NSW Australia; 3 Institute for Health and Sport, Victoria University, Melbourne, Australia

**Keywords:** Volunteer, Community sport, Motivations, Coach, Officiate

## Abstract

Sport participation and volunteering can make important contributions to good health. Sporting organisations need volunteers to deliver their participation opportunities and for many years the sector has faced challenges to volunteer recruitment and retention, especially due to the increased bureaucratic and compliance demands in operating community sports clubs. As sporting organisations pivot to adapt to COVID-safe sport we can learn about their experiences to inform volunteer recruitment and retention policies and practices. This research examined volunteer intentions and motivations in coaching and officiating in basketball and explored factors influencing their decision to return to COVID-safe basketball. Data was collected via an online survey that drew on theoretical frameworks of volunteer motivations (i.e. modified Volunteer Functions Inventory VFI) in sport as well as sport policies related to COVID-safe guidelines for return to sport. Data was collected in Victoria Australia during July 2020 before basketball had the chance to return from the first Australian-wide COVID-19 lockdown. Volunteers had positive intentions to return to basketball following COVID-19 restrictions because it was fun, to help others, or because friends/family were involved. Volunteers were most concerned that others will not comply with COVID-safe policies particularly around isolating when feeling unwell (95%), but also reported concerns about the inconveniences of some COVID-safe policies introduced to return to organised sport (e.g. social distancing, density limits, and enforcing rule changes). Understanding these volunteer intentions, motivations and factors influencing the decision to return to COVID-safe basketball can help inform recruitment and retention strategies to support volunteers in sport. Practical implications for sport policy and practice are discussed.

## Introduction

Sport participation has many health benefits and has been shown to contribute to Leisure-Time Physical Activity (LTPA) at health enhancing levels [1]. Systematic reviews found many psychological and social health benefits of participation in sport for children, adolescents and adults [2, 3]. Provision of sporting opportunities are important for contributing to active lifestyles and good health and many of these opportunities would not run without volunteers who fill critical roles such as coaches and officials. Volunteering has also been shown to contribute to better health outcomes and has been suggested as a public health strategy for a healthy lifestyle [4].

Volunteers are vital for community sport participation and the act of volunteering is defined as ‘time willingly given for the common good and without financial gain” [5]. In sport, volunteering is often defined under “non-playing roles…undertaken to support, arrange and/or run organised sport and physical activity” [6]. The most common non-playing roles in sport are coach and official followed by administrators and team managers [7], and other volunteer roles involve governance (e.g. board members). These roles make an important contribution to deliver organised sport in communities and provide positive sporting experiences. Around 3.1 million Australians participate in non-playing roles in sport and physical activity [7]. The majority of these non-playing roles are volunteers (2.9 million; 92%) with the peak age for volunteers being 35–54 years (48%) and nearly two-thirds were parents [7]. More men than women tend to volunteer in sport, with more men in coaching and officiating roles and women tending to fill administration and manager roles [8]. Others have also found similar demographics for sports volunteering especially being male and having children [9]. The authors suggest that the lack of women in volunteer roles was likely influenced by the gender bias in organized sports participation as volunteers are often recruited from within clubs [9].

Sporting organisations need volunteers to deliver their participation opportunities and for many years the sector has faced challenges to volunteer recruitment and retention, especially due to the increased bureaucratic and compliance demands in operating community sports clubs [10, 11]. It has been suggested that COVID-19 has exasperated the problems related to sport volunteering [12]. Sporting organisations in our society have not lived through extensive social disruption like we have experienced through the COVID-19 pandemic. Volunteers are often described as the backbone to organised sport and globally sporting organisations have had to pivot to adapt to ongoing changes to state and national guidelines for the return to play throughout multiple COVID-19 lockdowns and restriction changes. Government bodies have developed a suite of resources to support sporting organisations make a safe return to sport at all levels – including grassroots community participation [13, 14]. Many sport governing bodies have then developed these resources within the context of their specific sport in an effort to return to play in a safe environment [15]. This provides opportunity to learn from the COVID pandemic in terms volunteering in sport.

Research pre-COVID-19 had reported that volunteers in sport were already heavily burdened with increasing policy and legislative compliance in the day-to-day operations of sports clubs, which made it difficult to attract and retain volunteers in sport [16, 17]. A nationwide survey of Australian community sports clubs, across 20 sports, reported that 42% of clubs lost volunteers due to increased pressures and workload related to implementing COVID-19 protocols [18]. A recent study that explored the impact of COVID-19 on youth sport participation reported some of the difficulties and challenges that the COVID-19 lockdowns and subsequent reengagement in sport via COVID-safe policies had on volunteers and players returning to organised sport [19]. For example, coaches and players discussed the “protocols and hurdles” to run a junior sport program and feared the “additional layers of administration and organisation” may discourage some volunteers and players returning [19]. Research is yet to explore how the return to sport with COVID-safe policies and practices will impact on volunteers intentions to return or understand their concerns about complying to COVID-safe practises such as social distancing, enforcing rules to minimise unnecessary contact, and organising different models of training/competition to adhere to restrictions associated with venue capacity limits.

Basketball is a very popular sport in Australia and internationally for both women/girls and men/boys and as such requires a large workforce of volunteers. In Australia, as a result of the high number of grassroots participants, basketball appears in the top 3 volunteer sports for men and women [7]. Grassroots basketball in Australia is played year-round and indoors. The indoor nature of the sport meant that basketball was often faced with a greater level of COVID-19 restrictions than outdoor sports including longer lockdown/delayed return to sport, face masks, and indoor density limits. As we continue to live with COVID-19 in our communities we need to understand the impact of COVID on sport volunteers as it has likely exacerbated the administrative and operational workload of sport volunteers, which is a barrier to attracting and retaining volunteers in sport.

There is growing body of knowledge of impacts of COVID-19 on participation in sport and physical activity more broadly [19–21], and some reports on the impacts to community clubs [18]. Prior to the pandemic, a review of research found a lack of robust research to understand the needs, motivations and priorities of sports volunteers, especially coaches [22]. As community sport returns, we need to explore the intentions, motivations and concerns of volunteers and specifically coaches and officials who are relied on to deliver the sport and fulfill most non-playing roles. This information will help to strategically plan and develop appropriate sport policies at the local community level to ensure our volunteers are supported in a COVID-safe sport setting. This research, therefore aimed to understand:

1. What is the intention and motivations of coaches and officials to return to basketball?
2. What factors influence coaches and officials’ decision to return to COVID-safe basketball?

## Theoretical framework

Volunteer motivations in sport have commonly been examined from a sport event volunteering perspective [23], including mega-events [24, 25] but also sport-for development [26], coaching, officiating and administration [27, 28]. A systematic review of motivation of sport event volunteers found that most studies use quantitative methods, but nearly half of the studies had no specific theoretical measurement scales [23]. Understanding the psychology of volunteering is important as it identifies the factors influencing participation in voluntary roles. For example, understanding the motives for people to volunteer in sport can help to identify potential volunteers. Research on volunteer motivations have often been developed from functional theories in psychology, whereby motivational functions help explain the underlying reasons for engaging in volunteering [29]. The Volunteer Functions Inventory (VFI) is a widely used instrument for assessing volunteer motivations because of its theoretically sound basis and good psychometric properties [30]. The VFI aims to assess the motives to volunteer using six motivational functions (6 scale, 30 items) [31], which are cited below [29].

1. Values function: The opportunities that volunteerism provides for individuals to express values that are important to the self, related to altruistic and humanitarian concerns for others,
2. Understanding function: The opportunity for volunteers to gain and sustain knowledge, skills and abilities,
3. Social function: Volunteering offers opportunities to improve social relationships, it helps individuals to fit in and get along with social groups that are important for them,
4. Career function: Participation in voluntary work may increase future job opportunities,
5. Protective function: Participation in voluntary work can protect oneself from negative feelings about oneself, it can help the individual to eliminate negative aspects surrounding the ego and.
6. Enhancement function: By participation in voluntary work the individual can enhance his/her self-esteem, it centers on ego growth and development.

A systematic review of the VFI found that the “values function” (e.g. helping others) was higher than for all other motivations for both men and women, in volunteers under and over 40 years of age, and in all volunteer settings including health, social, education, sports, environment and civil defence [30]. For instance, “values” were the most important motivation function among youth sport volunteer coaches, but there were significant difference between first year and returning coaches for the functions of self-serving and personal growth which provided practical implications for recruitment and retention of youth sport coaches [27]. Similarly, Kim et al. [28] found motivational differences between youth sport settings, whereby volunteers in international youth sport events were highest in all six motivation factors followed by volunteers in special-needs events which were comparatively lower than volunteers in national and local youth sport organisations. The authors suggested that the prestigious, episodic and attractive opportunities for volunteering; particularly volunteering for philanthropic and humanitarian organisations (e.g. state-level Special Olympic event) likely influences volunteer motivations.

A modified version of the VFI has been used in youth sports, known as the Modified Volunteer Functions Inventory for Sport (MVFIS) which is a valid and reliable scale to measure volunteer motivations in youth sport [32]. The MVFIS has only 18 items, making it more efficient to administer than the VFI and is capable of measuring context-specific motivations of youth sport volunteers [32]. Researchers deemed this important as volunteers in youth sport programs have unique context-specific motivations compared to other volunteer settings such as parental involvement and attrition as children age [33]. Researchers have used the MVFIS to understand volunteer motivations across different youth sport organisations/events [28].

Researchers have reported a range of challenges to attract and retain volunteers in sport (club and events) at both an institutional level and an individual level [34]. Volunteers satisfied with the club’s working conditions (e.g. institutional level) and having children who belong to the club (e.g. individual level) have a positive influence on voluntary commitment [35]. Other factors that influence voluntary engagement include rurality whereby clubs in rural areas have more stable voluntary engagement independently of individual characteristics such as age, gender, competition experience [35].

## Method

To address the research questions, researchers partnered with a regional Basketball Association. A quantitative methodology was used, and questions relevant to this particular study were included in an online survey of registered players, coaches and officials. All research was conducted in accordance with human research ethics approval through Federation University, Victoria, Australia.

### Participants and procedures

The regional Basketball association had a membership size of 3652 registered players, that were predominantly male juniors (under 18 years) (43.0%). Registration of girls (26.4%), men aged 18+ (22.3%) and women (8.3%) were much lower. The online survey was advertised on the Basketball association’s webpage and social media platform (Facebook). In addition, all registered basketball players, coaches and officials aged 8 years and above were sent an email invitation to the online survey by the basketball association. The invitation was sent (27th July 2020) as the second wave of COVID infections was emerging in Victoria and the metropolitan area was locked down [36]. Regional Victoria later entered a second stage 3 lockdown on 5th August 2020 [37] and the survey was closed on the 10th August, 2020). A total of 344 respondents completed the survey, of whom 75 met the inclusion criteria for this particular study of coaches and officials. The majority (97%) of survey responses were completed within the first 5 days (27-31st July 2020). At this point in time, study participants had not been able to return to basketball since the nationwide lockdown.

The basketball association was the ethical gatekeeper for the privacy of the study participants and the researchers did not have access to any participant names or contact details. The invitation was tailored to registered players, coaches and officials from the 2019 and 2020 seasons. The emailed invitation included a link to the online survey which first provided a plain language statement and informed consent. Children aged 17 years or under, required parent (or caregiver) consent; whilst children under 13 years old required their parent/caregiver to help complete the survey. Participants were not able to start the survey unless they (and their parents where applicable) gave their consent.

### Survey instrument

The online survey was developed in consultation with the Basketball Association to collect data on players and volunteers’ intentions, motivations, and concerns and the impact of COVID-19 in returning to sport following COVID-19 lockdowns and restrictions. The development of survey questions drew on theoretical frameworks of volunteer motivations in sport and specifically the VFI used in youth sports [31, 32]. The VFI [31] was modified by the first author to reduce the number of items within each of the six functions to minimise respondent burden in completing the survey that measured several key areas. The final list of volunteer motivations were reviewed and refined by the research team in collaboration with the Basketball Association, who formed the expert group to verify the construct and content validity. This included a total of 14 motivations, outlined below, and included two new items that did fit existing functional areas (e.g. There is some reimbursement or hobby income, and fun and enjoyment). The items for volunteer motivations are listed below and best aligned with the interests of the Basketball Association.

1. Values: I feel it is important to help others.
2. Understanding: It allows me to explore my own strengths; I can learn how to deal with a variety of people; To learn a new skill/expand on knowledge of the game.
3. Social: Friends or family are involved in basketball; People want or ask me to coach or officiate basketball.
4. Career: It assists me to develop my career.
5. Protective function: Coaching/officiating in basketball helps me work through own personal problems; Coaching/officiating in basketball is a good escape from my own troubles; Psychological/mental health/therapy; Physical health or fitness.
6. Enhancement function: It is a way to help me make new friends.
7. Other: There is some reimbursement or hobby income; Fun and enjoyment.

The development of survey questions also drew on relevant literature and sport policies including physical activity measured in line with the Australian Government’s physical activity guidelines [38], and COVID safe guidelines for the return to sport [15, 39]. The survey contained questions relating to five key areas and survey question branching was used so that respondents only answered questions relevant to them. This study only reports responses of participants who identified as a volunteer in either a coach or officiating role, although some of these participants also ‘played’ basketball. The online survey was pilot tested by co-authors and several staff at the basketball association to ensure relevance and online functionality. Minor wording changes were made. The survey was took approximately 10 min to complete and included the following question categories:

1. **Demographics**: Date of birth, Residential postcode, Gender, Education, Employment status, Household type (members), Aboriginal or Torres Strait Islander status, Language other than English spoken at home.
2. **Organised sport and physical activity profile**: registered sport (e.g. basketball) participant, coach or official and other organised sports participated in 2019 and 2020, meeting physical activity guidelines [38].
3. **Motivations**: reasons for participating, coaching, or officiating basketball last year and when returning to basketball following COVID-19 [32].
4. **Intentions to return to playing, coaching, officiating**: I do not intend to return (i.e. drop out), I intend to return, but not until next year, I intend/hope to return later this year, I have returned to training/practice only, I have returned to competition (i.e. retained groups), and undecided/unsure.
5. **Factors influencing decision whether to return to sport**: Health and safety concerns relating to COVID-19 about returning to basketball, Inconveniences of guidelines to return to basketball, impact of COVID-19 on coaching and officiating, confidence in everyone’s compliance with government COVID safe guidelines and restrictions [15, 39].

### Data analysis

Survey data was collected using Qualtrics and then transferred to STATA for cleaning & analysis. Analysis for this paper was limited to those who indicated they were involved in basketball as a coach or official and intended to return in either capacity. The multiple response items for reasons for participating were crosstabulated, displaying the number and percentage of all respondents in two age cohorts (adolescents and adults) who selected each reason. Responses to Likert scale items for concerns about health & safety and inconvenience were recoded from a three-point scale (not concerned, somewhat concerned, concerned) to a dichotomy (not concerned or concerned). Responses to Likert scale items for impact of COVID-19 on volunteering and confidence of basketball complying with government COVID safe guidelines were recoded from 5 categories (strongly disagree, disagree, neither disagree or agree, agree, strongly agree) to three categories (strongly disagree/disagree, neither disagree or agree, agree/strongly agree). Chi square tests of differences of proportions, and Fishers exact tests where appropriate because of small cell counts, were used to determine statistical significance of the differences between the responses of adolescents and adults for commitment to returning, motivations, concerns, impacts and confidence.

## Results

A total of 344 respondents completed the survey, of whom 75 met the inclusion criteria for this particular study of coaches and officials. Many of these were also players (n = 50; 66.7%) and some had multiple volunteer roles (n = 14; 18.7%). The total numbers of responses listed in Tables 1, 2, 3, 4 and 5 differ because of these 14 respondents who were both coaches and officials. Table 1 reports coaches and officials separately so the 14 responses were double counted, leading to a total of 89 responses. Of these, 82 responses indicated an intention to return. The number of respondents intending to return was 14 less than this, or 68, as shown in Table 2. Tables 3, 4 and 5 are based on the 75 respondents, with no double counting.

**Table 1:** Intention to return to volunteer roles among basketball volunteers: overall and by age group

	All respondents		Adults 18+		Adolescents 13–17 yrs		p-value
Volunteer role	Count	%	Count	%	Count	%	
**Coach**							
Total number of coaches	52		44		8		
Committed to returning	46	88.5	38	86.5	8	100.0	0.573
**Officials**							
Total number of officials	37		27		8		
Committed to returning	36	97.3	28	96.5	8	100.0	0.784

**Table 2:** The motivations of volunteers who were committed to returning after COVID lockdown: overall and by age group

	All respondents (n = 68)		Adults 18+ (n = 55)		Adolescents 13–17 yrs (n = 13)		p-value
Motivations	Count	%	Count	%	Count	%	
Fun/enjoyment	49	72.1	38	69.1	11	84.6	0.198
Friends or family are involved in basketball	43	63.2	36	65.5	7	53.9	0.507
I feel it is important to help others	42	61.8	34	61.8	8	61.5	0.764
People want or ask me to coach or officiate basketball	34	50.0	29	52.7	5	38.5	0.761
To learn a new skill/expand on knowledge of the game	22	32.4	14	25.5	8	**61.5**	0.015
I can learn how to deal with a variety of people	18	26.5	12	21.8	6	46.2	0.069
Physical health or fitness	17	25.0	11	20.0	6	46.2	0.062
There is some reimbursement or hobby income	14	20.6	8	14.6	6	**46.2**	0.012
It allows me to explore my own strengths	14	20.6	6	10.9	8	**61.5**	< 0.001
Psychological/mental health/therapy	14	20.6	12	21.8	2	15.4	0.545
It assists me to develop my career	11	16.2	5	9.1	6	**46.2**	0.002
It is a way to help me make new friends	11	16.2	6	10.9	5	**38.5**	0.019
Coaching/officiating in basketball is a good escape from my own troubles	8	11.8	5	9.1	3	23.1	0.136
Coaching/officiating in basketball helps me work through own personal problems	9	13.2	5	9.1	4	**30.8**	0.043

**Table 3:** Volunteers’ concern about returning to coach and/or volunteer basketball: overall and by age group

	All respondents (n = 75)		Adults 18+ (n = 62)		Adolescents 13–17 yrs (n = 13)		p-value
Concerns	Count	%	Count	%	Count	%	
**Health and safety concerns**							
Others will not stay home or self-isolate when they are feeling unwell	71	94.7	59	95.2	12	92.3	0.541
Others will not stay home or self-isolate when they have COVID-19 infection	69	92.0	56	90.3	13	100.0	0.582
Risk of COVID-19 infection from asymptomatic (no symptoms) individuals	69	92.0	58	93.5	11	84.6	0.277
The hygiene practices of others (e.g. washing hands, hand sanitizer use, minimize touching of face)	64	85.3	52	83.9	12	92.3	0.677
Risk that shared equipment will not be sanitized/cleaned appropriately between sessions	45	60.0	37	59.7	8	61.5	0.579
Risk that the facilities will not be sanitized/cleaned appropriately everyday	42	56.0	36	58.1	6	46.2	0.543
Members of my family and/or household have underlying health issues	36	48.0	29	46.8	7	53.8	0.763
I have underlying health issues	12	16.0	7	11.3	5	**38.5**	0.029
**Concerns about the inconveniences in returning to sport**							
A second COVID-19 wave is likely to shut sport down again	68	90.7	55	88.7	13	100.0	0.343
Social distancing requirements in the venue	39	52.7	31	50.8	8	61.5	0.552
Other family members will not be able to attend the basketball venue with me	37	49.3	29	46.8	8	61.5	0.375
Organising training schedules under the new COVID-19 basketball guidelines	35	46.7	26	41.9	9	69.2	0.124
The need to use hand sanitizers courtside when a player enters and exits the court at breaks, timeouts and substitutions	21	28.0	15	24.2	6	46.2	0.171
I will be unable to shower or change at the venue	7	9.3	3	4.8	4	**30.8**	0.015
Organising different models of competition (format, scheduling, timing, rules)	33	44.0	24	38.7	9	69.2	0.065
Enforcement of rule changes to minimise unnecessary contact (e.g. hand shakes, high fives, pushing off the ball)	32	42.7	26	41.9	6	46.2	0.507
Taking on new roles or responsibilities such as COVID-19 bio-safety officer	29	38.7	24	38.7	5	38.5	0.622

**Table 4:** Impact of COVID-19 on volunteers: by age group

	Adults 18+ (n = 61)			Adolescents 13–17 yrs (n = 13)			p-value
	Disagree	Neither disagree or agree	Agree	Disagree	Neither disagree or agree	Agree	
Impacts	%	%	%	%	%	%	
I have taken time during COVID-19 to improve myself as a coach/official	54.1	23.0	23.0	38.5	38.5	23.1	0.476
The resources published by basketball governing bodies have been useful for developing my coaching/officiating	23	24.6	52.5	7.7	53.8	38.5	0.121
The club/organisation has supported me during COVID-19	19.7	21.3	59.0	15.4	30.8	53.8	0.769
I am motivated to return to my coaching/officiating role	26.2	16.4	57.4	7.7	23.1	69.2	0.398
My weekly routine (home, work or study) has changed and I now lack time for coaching/officiating	75.4	13.1	11.5	69.2	30.8	0.0	0.245
I have enjoyed finding new things to do with my time, rather than coaching/officiating basketball	47.5	16.4	36.1	**61.5**	38.5	0.0	0.010
I have chosen to coach/officiate in other organised sports	77	9.8	13.1	76.9	15.4	7.7	0.754
My friends are not returning to basketball, so I am not returning to coach/officiate	86.9	13.1	0	92.3	7.7	0.0	0.503
Other family members are not returning to basketball, so I am not returning to coach/officiate	86.9	8.2	4.9	100.0	0.0	0.0	0.765
Basketball coaching/officiating is not a priority for me at the moment	57.4	13.1	29.5	76.9	23.1	0.0	0.052
It is not worth returning coaching/officiating until there are fewer restrictions in place	59	19.7	21.3	92.3	7.7	0.0	0.069

**Table 5:** Confidence in everyone’s compliance with government COVID safe guidelines and restrictions: by age group

	Adults 18+ (n = 62)			Adolescents 13–17 yrs (n = 13)			p-value
	Disagree	Neither disagree or agree	Agree	Disagree	Neither disagree or agree	Agree	
	%	%	%	%	%	%	
I have confidence in basketball **venue operators** to implement COVID-19 safe guidelines to ensure a safe return to sport and compliance with government restrictions.	11.3	9.7	79.0	0.0	7.7	92.3	0.629
I have confidence in the **basketball association** to implement Basketball Australia’s COVID-19 safe guidelines to ensure a safe return to sport and compliance with government restrictions.	11.3	9.7	79.0	0.0	7.7	92.3	0.629
I have confidence in **basketball clubs** to implement COVID-19 safe guidelines to ensure a safe return to sport and compliance with government restrictions.	9.7	11.3	79.0	0.0	15.4	84.6	0.626
I have confidence that **basketball players** will adhere to the guidelines and restrictions.	30.6	27.4	41.9	23.1	30.8	46.2	0.861
I have confidence **in myself** to adhere to the guidelines and restrictions whilst playing other sports.	0.0	1.6	98.4	0.0	7.7	92.3	0.319

Table 1 shows the intention to return to basketball coaching and/or officiating roles when COVID-19 restrictions eased. Nearly all volunteer roles had commitment from their respective coach and official to return for both adolescents (100%) and adults (92.1%).

Table 2 shows that volunteer motivations to return to basketball when COVID-19 restrictions eased were primarily related to “fun and enjoyment” the individuals received from volunteering among both adults (69.1%) and adolescents (84.6%). Volunteers were also motivated because they felt “it was important to help others” (adults 61.8% and adolescents 61.5%). Adolescents were significantly more likely than adults to report motivations to “learn a new skill/expand on knowledge of the game” (61.5%, 32.4%, p = 0.015), “it allows me to explore my own strengths” (61.5%, 20.6%, p < 0.001), “it assists me to develop my career” (46.2%, 9.1%, p = 0.002), “helps me to work though own personal problems” (30.8%, 9.1%, p = 0.043), “it is a way to help me make new friends” (38.5%, 10.9%, p = 0.019), and “there is some reimbursement or hobby income” (46.2%, 20.6%, p = 0.012). Whilst not significant, adult volunteers reported motivations relating to “friends and family are involved in basketball” (65.5% v 53.9%) and “people want or ask me to coach or officiate basketball” (52.7% v 38.5%).

Volunteers’ main concerns about returning to basketball were that others will not stay home or self-isolate when they are feeling unwell (adults: 95.2%; adolescents 92.3%) or have COVID-19 infection (adults: 90.3%; adolescents 100.0%). Table 3 shows volunteers were also concerned that they risked COVID-19 infection from asymptomatic individuals (adults: 93.5%; adolescents 84.6%). Adolescents were significantly more likely to report being concerned about returning because they had underlying health issues (38.5%; 16.0%, p < 0.05).

Volunteer concerns about the inconveniences of COVID-safe policies introduced to return to organised sport safely were primarily related to the likelihood that a subsequent COVID-19 wave of infections would shut the sport down again (adults: 88.7%; adolescents: 100.0%), followed social distancing requirements in the venue (adults: 50.8%; adolescents: 61.5%) and other family members unable to attend the venue with volunteers (adults: 46.8%; adolescents: 61.5%). Many volunteers were also concerned about many of the logistical and operational changes required to return to sport such as organising training schedules under the new COVID-19 basketball guidelines (adults: 41.9%; adolescents: 69.2%) and organising different models of competition (adults: 38.7%; adolescents:69.2%). Adolescents were significantly more likely to report being concerned about being unable to shower or change at the venue (30.8%; 4.8%, p < 0.05).

The impact of COVID-19 on volunteers is presented in Table 4. Most volunteers disagreed that family (adults: 86.9%; adolescents: 100.0%) and friends (adults: 86.9%; adolescents: 92.3%) were not returning to basketball. Positively, most volunteers agreed that they were motivated to return to their coaching/officiating role (adults: 57.4%; adolescents: 69.2%), their club/organisation had supported them during COVID-19 (adults: 59.0%; adolescents: 53.8%), and the resources published by basketball governing bodies were useful for developing coaching/officiating (adults: 52.5%; adolescents: 38.5%). Almost a quarter of volunteers agreed with the statement about taking time during COVID-19 to improve myself as a coach/official (adults: 23.0%; adolescents: 23.11%). Adolescents were significantly more likely to disagree with the statement that they have enjoyed finding new things to do with their time, rather than coach or officiate basketball (61.5%, 47.5%, p < 0.05).

Table 5 presents the volunteers’ confidence in everyone complying with government COVID safe guidelines and restrictions. The majority of volunteers agreed that they had confidence in the basketball venue operators, association, and clubs to implement COVID-19 safe guidelines to ensure a safe return to sport and compliance with government restrictions (range: 79.0 − 92.3%). Some volunteers had less confident in basketball players adhering to guidelines and restrictions, with small percentage of adults (41.9%) and adolescents (46.2%) agreeing that basketball players will adhere to the guidelines and restrictions.

## Discussion

The pandemic caused unprecedent social disruption to daily living including community sport and the ‘backbone’ of the industry, its volunteer workforce. Volunteers are vital to community sport fulfilling various roles particularly at grassroots. They are coaches and officials, administrators, event organisers fundraisers and more. However, volunteers in sport are often burdened with increasing policy and legislative compliance in the day-today operations of sports clubs, which makes it difficult to attract and retain them [16, 17]. Emerging research has reported fears that the additional protocols related to the administration and organisation of COVID-safe sport may discourage volunteers returning [19]. This research sought to understand volunteer coaches and officials’ intentions, motivations and concerns about returning to basketball following COVID-19 disruptions.

We found volunteers had positive intentions to return to sport following COVID-19 restrictions. There has been a strong desire among various communities to return to normal life, especially for our children and adolescents and via COVID-19 vaccination [40]. In Australia, there has been high uptake in vaccination among adults and many parents have already vaccinated their children, or intend to, as vaccination rolls out to younger age groups [41]. Many volunteers in sport are also parents [7] and likely want to see the return of sport so that their children can enjoy playing and reap the many personal and health benefits of participation. As demonstrated in this study sports volunteers were motivated to return to coaching or officiating basketball because they felt it was important to help others and to support friends and family that play.

As society emerges from COVID-19 restrictions, and lives with COVID-19 in our communities it is important to appeal to the motivations of volunteers and in particular appeal to different cohorts of volunteers and their underlying motivations as this may help to attract and recruit the volunteer workforce. In our study we found that the number one reason both adolescents and adults were motivated to return to coaching or officiating basketball was because it was fun and/or enjoyable. Fun and enjoyment have been previously reported as a reason for volunteering in sport [42]; although is not typically included on the VFI [32]. Others have commented that “people volunteer for various and complex reasons; thus it is too simplistic to understand volunteer motivation with a single concept or theory” [43] as cited in Kim et al. 2010b. The main motivation for volunteering in community sport mirrors that for participants, they volunteer and play for fun and enjoyment [44–46]. These motivations are different to volunteers in other sectors. For instance, public health volunteers common motives were about supporting a cause, helping others, or civic duty [47].

Other motivations that were reported tended to be somewhat different for adolescents compared to adults and highlights motivations likely change across the lifespan. Adolescents were motivated by “development” motivations to explore their own strengths, learn a new skill, expand on knowledge of the game, or develop their career. In comparison, adults cited “social” motivations as friends or family were involved with basketball or people want or ask me to coach or officiate basketball. Studies of volunteer motivations in sport and recreation setting is sparse, but some previous research has reported that teenage volunteers place more importance on motivations related to understanding, career and values motivations [28]. Further analyses could be undertaken to identify volunteer motivations by gender, but this is beyond the scope of this paper; particularly as “motivations” were not the sole focus of the research.

This research also sought to understand factors influencing volunteers’ decision to return to COVID safe sport. The majority of volunteers reported concerns related to the perception that ‘others’ will not stay home or self-isolate when they are feeling unwell or have COVID-19 infection. There was also a range of health and safety concerns relating to hygiene practices and sanitizing or cleaning shared equipment and facilities. The emergence of COVID-19 has led to a range of fears, worries and anxiety across the globe [48] and research has focused on understand individual’s distress and fear to inform interventions to support mental wellbeing [49]. Considering that volunteers in sport are worried about ‘others’ and in particular basketball ‘players’ complying with COVID-safe protocols it is important that sport administrators provide their volunteers with a degree of confidence that a COVID-safe environment will be monitored, and policies adhered to ensure safe operation. Hooker and Leask [50] discussed the consequences of failure to communicate early during the first stage of the COVID-19 pandemic in Australia and the United Kingdom. They suggested principles of risk communication; in particular early and proactive communication (e.g. early and often) might have supported “earlier sense-making, encouraged citizen engagement and provided potential convergence towards a response strategy” (p.3–4). Whilst this analysis is primarily in relation to public and government communication there may learnings for sport particularly to adopt risk communication strategies that do not dismiss concerns and provide some sense of control as individuals navigate concern and uncertainty. Therefore, sport governing bodies can support their affiliated associations and clubs with appropriate risk communication policies and resources particularly around compliance with COVID-safe protocols.

Research [51] in the hospitality sector has investigated how organizations can facilitate employees’ compliance with COVID-safe health and safety regulations. They found that compliance is driven by management safety practices and organization response strategies prioritizing safety. Whilst we recognise these findings are interpreted in the context of hospitality industry and a Chinese culture there may be some learnings that can be applied by sport administrators to enhance volunteer perceptions and confidence of a COVID-safe environment. For instance, Hu et al., (2021) found that compliance is not static, requiring continuous practice of safety behaviours as individuals revise their perceptions of risks and safety procedures. Perceptions of risks and safety are likely to change as vaccination rates increase and COVID-19 case numbers decline. Hu et al., [51] also reported that genuine workplace commitment to safety can be achieved through three management practices (1) Protecting (e.g. decision-making based on safety concerns and provision of safety resources); (2) Promoting (e.g. communicating importance of safety via meetings etc.); and (3) Participating (e.g. top-down guidance and safety audits, as well as bottom-up involvement). Future studies could verify and extend these findings in the Australian sport and volunteer context. It is likely that multi-level strategies that not only focus on individuals and their behaviour (volunteers, players, spectators, parents) but also organisational policies and strategies are required to alleviate health and safety concerns whilst providing a safe environment. This is important considering that in addition to these health and safety concerns, around half of volunteers reported being concerned about the range of inconveniences in returning to sport such as social distancing requirements, density limits, and logistics of organising training schedules or competitions under new COVID-19 guidelines. Enforcing rule changes to minimise unnecessary contact and taking on new roles or responsibilities such as COVID-19 bio-safety officer were also reported by over a third of volunteers. Many of these concerns may change as vaccination rates increase and COVID-19 case numbers decline, although new variants may also emerge and as such there is likely continued worry/anxiety about transmission for some time.

Finally, in terms of the impact of COVID-19 on volunteers, albeit these were collected early in the pandemic, there were few negative impacts reported with many disagreeing with statements that family or friends were not returning; that they lacked time for coaching/officiating because of the impact of COVID-19 on weekly routines; or that it was not worth returning to coaching/officiating until there are fewer restrictions in place. Positively, many volunteers reported feeling supported by their club or organisation during COVID-19 and many found the resources published by the governing body as useful for developing their coaching/officiating. Almost a quarter of volunteers took time to improve themselves as a coach/official (23%). This demonstrates the importance of developing and providing volunteers with resources, especially during lockdowns as a strategy to keep volunteers engaged in sport.

## Limitations and future implications

This research is the first to explore the intentions and motivations of coaches and officials to return to basketball once COVID-19 restrictions were lifted. It also investigated factors influencing their decision with COVID-safe protocols. A limitation of this study was that the online survey was only distributed at one time point, between Wave 1 and Wave 2 of COVID-19 infections in Victoria Australia and before the sport of basketball was able to resume in Victoria. More longitudinal research is needed to monitor volunteering rates in sport, as well as volunteer motivations and concerns to inform recruitment and retention strategies. This is important as COVID-19 is not static and perceptions of risks and safety may change, especially with vaccination uptake and new variants emerging. There may also be a sampling bias whereby individuals who were motivated to return to basketball may be more likely to take part in this research. The self-report survey approach was cost effective to capture a large participant base during a time when sport participation and subsequent volunteering was shutdown.

## Conclusion

COVID-19 and associated restrictions do not appear to have severely negatively impacted volunteer coaches and officials’ intentions to return to basketball. This research identified a range of practical implications for sport policy and practice. First, many volunteers were motivated to return to basketball because it was fun/enjoyable, it was important to help others and because friends and family were involved in basketball. Understanding these motivations can help inform recruitment and retention strategies. Sport administrators and governing bodies could tap into the community’s desire to return to normal life and emphasise how getting back to sport, both playing and volunteering, is part of life. Second, it will be crucial to develop and continually refine multi-level strategies that not only focus on individuals and their behaviour (volunteers, players, spectators, parents) but also organisational policies and strategies to alleviate health and safety concerns related to COVID-safe basketball and support volunteers in the return to sport. Finally, this research demonstrated the importance of developing and providing resources to volunteers to develop and/or improve their coaching or officiating. This may be an important strategy to keep volunteers engaged in sport, especially during forced absence of community sport.

## Data Availability

The datasets generated and/or analysed during the study are not publicly available but are available from the corresponding author on reasonable request.
